# Ambulatory pediatricians: how to bridge the gaps in diagnosis and care coordination for neurodevelopmental disorders in France

**DOI:** 10.3389/fped.2024.1269198

**Published:** 2024-04-25

**Authors:** Thiébaut-Noël Willig, Jean-Paul Blanc, Rémy Assathiany, Claire Bilbault, Laurent Raffier, Andreas Werner

**Affiliations:** ^1^ Clinique Ambroise Paré, ELSAN Group, & Eventail 31, Toulouse, France; ^2^ Occitadys, Toulouse, France; ^3^ Association Française de Pédiatrie Ambulatoire (AFPA), Ancenis Saint-Géréon, France; ^4^ Association Française des Neuropédiatres Libéraux (AFNL), Chatenay Malabry, France; ^5^ CHR Metz Thionville, Ars Laquenexy, France

**Keywords:** neurodevelopmental disorders, healthcare pathways, healthcare organization, private medicine, child

## Abstract

**Introduction and aims:**

The organization of healthcare pathways for neurodevelopmental disorders (NDD) relies on different levels of expertise depending on the complexity of these disorders. NDDs affect between 8% and 15% of children. Historically, national recommendations and healthcare planning measures were initially devoted to autism spectrum disorders and were gradually extended to Attention deficit hyperactivity disorder (ADHD) and specific learning and development disorders. Private doctors play an increasing role in these pathways at different levels of care due to difficulties in organization, particularly in the health and social sector. The aim of this work was to evaluate the contribution of second-line private doctors in the diagnosis and care of children affected by NDD.

**Methods:**

A first series of surveys in 2016 evaluated the level of commitment of primary care pediatricians; this online national survey was repeated in 2023 among 1,430 members of the French Association of Ambulatory Pediatrics (Association Française de Pédiatrie Ambulatoire: AFPA) to assess their training, current and future involvement, and activity in NDD care. Analysis was performed by the main author using Epi-Info software.

**Results:**

The study identified in 2023 214 second-line private doctors (14% of all pediatricians in activity), of which 185 agreed to appear in a directory published the same year by the AFPA to facilitate referrals from other professionals. Sex ratio of responders is usual for paediatricians: 79.5%/20.5% (F/M), with a distribution among ages showing a slight increase of the age range between age 51–60 (30.5%). Our data indicate that in France in 2022, second-line private doctors made 48%–53% of NDD diagnoses, 24%–26.4% of follow-up consultations and declare to be accountable for 21% of initial prescriptions for Methylphenidate. Among these second-line doctors, 40% had completed a post-university degree on NDD, 74.3% had completed professional development training (PDT) and 85.2% had completed either or both types of training. Most doctors participating in the survey wanted to improve their level of practice, suggesting that in five years, the number of second-line private doctors will increase by 20% to 244 despite 24 planned retirements within the same period. This data probably underestimates the role of private doctors in NDD diagnosis, follow-up, and initial Methylphenidate prescriptions given the unfavourable working conditions (no financial compensation for long appointments, difficulty accessing paramedical and psychological assessments).

**Conclusions:**

Our data confirms that diagnosis and care coordination in the various presentations of NDD may rely on different types of practices and specializations: medical and social professionals, mental health professionals, but also a growing body of medical doctors involved in developmental and behavioural pediatrics. This data and reflection will be helpful for organizing healthcare in France or in other countries. Main study limitation relies in the self-declaration of MD's involvement in NDD and could not evaluate the activity of employed MD's from the social and medico social sector, nor be based on the national databases for prescription. It remains however the first attempt of characterization of medical activity at the national level in France for NDD.

## Introduction

In France, disparities persist in accessing care pathways for children with neurodevelopmental disorders (NDD), influenced by territorial disparities, socioeconomic status, and the availability of trained practitioners. NDDs, affecting 8%–15% of children globally [for references (1):], encompass conditions such as autism spectrum disorders (ASD), attention deficit hyperactivity disorder (ADHD), specific language impairment (SLI), developmental coordination disorders (DCD), specific learning disorders (SpLD), and intellectual developmental disorders (IDD). These conditions often overlap (2), complicating diagnosis and necessitating multidisciplinary evaluations for tailored interventions (3, 4), while simple conditions might also benefit from single practitioners (5).

France has implemented national strategies and recommendations, focusing on autism (6), IDD (7), ADHD (8), language, learning (9) and motor disorders (10) together with the early detection of children at risk for NDDs (11), prefiguring the unification of the different disorders onto the paradigm of NDD in a national recommendation.

However, historical fragmentation in healthcare services has hindered integrated care delivery. Initiatives such as national coordination platforms for early diagnosis and intervention (Plateformes de coordination et d'orientation: PCO) have been introduced (12), albeit with limitations in individual medical assessments.

The healthcare landscape in France includes mental health units, health and social units, reference centers, and private or hospital-based practitioners.

A first national study conducted by the French Association of Ambulatory Pediatrics (AFPA) in 2016 specified the role of primary care pediatricians in France (1, 13), describing the levels of practice and competence of these doctors. According to the results of this survey, their training was mainly based on post-university training within the framework of university degrees, continuing professional development (CPD) (Développement Professionnel Continu: DPC) or funded by a training insurance fund (Fond d'Assurance Formation: FAF). A second, more comprehensive survey in 2021 described the medical services for child ADHD offered in two regions: Auvergne-Rhône-Alpes and Occitanie (14).

The increase in the skills and number of pediatricians getting involved in neurodevelopmental disorders is faced with a lack of visibility and recognition by regional and national authorities; there is also insufficient financial recognition for these time-consuming consultations, limiting this activity for private practitioners in sector 1 (sector with a fixed price for medical doctors). To overcome this barrier, the TSLA Occitanie pathway [Parcours de Santé Troubles Spécifiques du Langage et des Apprentissages (TSLA) Occitanie] was set up on an experimental basis and offers a solution through a care financing package for second-line doctors (15), together with the structuration of first and second line structures.

This study aims to provide updated insights into the French NDD healthcare landscape to inform policy developments and international collaborations, specifically on the role of private practitioners. Through an examination of historical context and current challenges, we seek to contribute to improved care provision for children with NDDs in France and beyond.

## Materials and methods

### Design and diffusion of the study

From November 2022 to January 2023, through an initial mailing to members of the AFPA, networks of care in the Normandy, Auvergne-Rhône-Alpes, and Occitanie regions and two scientific societies of pediatrics (AFPA, SFNP), we identified the ambulatory physicians practicing in second-line care for NDDs. Participation was also proposed to representative associations of child psychiatrists and other care networks for NDDs/SpLD disorders. The aim of this first step was to establish a national directory of second-line independent physicians while respecting the General Data Protection Regulation (GDPR).

In the second phase, in February and March 2023, we asked participating physicians to describe their practice methods, specialty, training and diagnostic activity using the Google Forms software, without collecting personal data or IP addresses. The questions pertained to demographic data (8 questions) their training about NDD (7 questions), including continuous education needs, their current involvement on NDD including the organization of their practice. Questions investigated the monthly number of diagnoses and follow-up consultations for NDDs (16 questions) and the initial prescription of Methylphenidate in child and adolescent attention/hyperactivity deficit disorder (ADHD) in 2022.

### Level of care of responders

As in previous surveys, they were asked to place themselves at one of three levels of care for the diagnosis and management of SpLD disorders at the time of the survey and in five years (two questions), and their estimation for retirement's year and discontinuation of their specialized activity (9, 15).

Level 1 (first line): detection, screening, analysis, and validation of complaints, prescription, and interpretation of assessments in simple situations, initial response proposals (re-education, educational adaptations, advice), and referral to level 2 if necessary.

Level 2 (second line): referral in complex situations involving multiple associated disorders, which require the coordination of multidisciplinary assessments, prioritization (physicians practicing specialized consultations on developmental and learning disorders), and follow-up.

Level 3 (third line): expertise in complex situations, such as genetic syndromes with specific neurocognitive profiles, complex epilepsy, failed rehabilitation, or situations requiring specialized multidisciplinary teams (reference centers).

A fourth option: “Physician untrained or not oriented on these issues” was also available.

The survey with the link to fill out the questionnaire sent by email was conducted in two waves among second-line physicians and the 1,430 pediatricians of the AFPA in February and March 2023.

Ethics: No personal data were collected or analysed, and each of the associations that distributed the participation link for the study ensured compliance with the GDPR (General Data Protection Regulation).

### Statistical analysis

A statistical analysis was performed by the first author using an Excel spreadsheet containing all data and the Epi Info Version 7.2.2.6 software distributed by Centers for Disease Control and Prevention (CDC) (16).

Descriptive statistics were generated from the software and mean comparison was performed using the ANOVA technique in case of homogeneity of variance between groups, or otherwise by the Kruskal-Wallis test for two groups.

### Reference values for epidemiology and methylphenidate prescription

The reference prevalence for NDDs was chosen at 8% based on usual data resulting from the international literature (1, 17), the average annual birth rate in France at 812,000 according to INSEE data (18).

The prevalence of Methylphenidate prescription reported in published international data (19, 20). Regarding Methylphenidate prescriptions, we divided the respondent population into four profiles: no first prescription, “occasional” first prescription defined as monthly or less, up to one prescription a week, and more than one prescription a week (strong first prescription activity).

## Results

### Demography

Out of the 395 responses obtained, 374 questionnaires were retained for the study due to duplicate entries, resulting in a response rate of 26.1% based on the total number of members of AFPA (1,430 at the time of the survey). The participating private practice doctors were distributed across all French regions including overseas departments and territories. The gender ratio matched that of the population of pediatricians of 79.5/20.5 (female/male).

The distribution by age showed good representation of all age groups, with a slightly higher participation rate in the 51–60 age group (30.5% of responses) (Table 1).

**Table 1:** Distribution in the level of care in 2023 based on the age of the physicians, their studies and where they work.

In 2023	Not involved	Level 1	Level 2	Level 3	Total
**By age (*n* = 374)**					
< 31 years old			(1)		1
31–40 years old	3.7% (3)	48.1% (39)	46.9% (38)	(1)	81
41–50 years old	2.1% (2)	47.4% (46)	50.5% (49)	(0)	97
51–60 years old	3.5% (4)	35.9% (41)	58.8% (67)	(2)	114
61–70 years old	2.7% (2)	27.4% (20)	68.5% (50)	(2)	73
> 70 years old		(3)	(5)		8
**Profession (*n* = 374)**					
Pediatrician	3.2% (11)	43.6% (147)	52.5% (177)	0.6% (2)	337
Pediatric neurologist	(0)	(0)	95% (19)	5% (1)	20
Child psychiatrist	(0)	(0)	83.3% (5)	16.7% (1)	6
General practitioner	(0)	18.2% (2)	81.8% (9)	(0)	11
Total	11	149	210	4	374
After 5 years: not involved or level 1	90		3		83
After 5 years: level 2 or 3	69		188		257
After 5 years: retired	11		23		34

Data displayed as a percentage, between brackets: number of respondents.

The number of private practice doctors who responded was 210 in second line and 4 in third line, for a total of 214, distributed as follows: 199 pediatricians (including 20 neuropediatricians), 6 child psychiatrists and 9 general practitioners (GPs).

Half of the pediatricians in first-line care in 2023 wished to acquire a level of competence in second line care within the next five years. According to projections, the 23 second- or third-line doctors who plan to retire in the coming years would be replaced by the 69 doctors who wish to get trained within the next 5 years. The number of second- and third-line doctors would be 254 and 3, respectively, representing a 20% increase compared to the current situation (Table 1).

An additional analysis was conducted to determine if the ability to conduct consultations with fee overruns or a specific flat-rate pricing (TSLA Occitanie healthcare pathway, without fee overruns) influenced the activity of doctors in neurodevelopmental disorders. For second-line doctors, whose diagnostic evaluation consultations usually last at least one hour, the frequency of diagnosis of NDDs (more than once a week) depended on the possibility of this financial valorisation (Kruskal–Wallis test *p*: 0.03), which was not the case for first-line doctors with shorter times of consultations.

### Training

Overall, 28.6% of respondents have received a university degree in the field (Table 2) (40% among second-line doctors, 100% for third-line doctors). CPD/DPC training, an alternative to degree programs, was often chosen by private practice doctors as a supplement (66.3%), with 74.3% of second-line doctors and 57.7% of first-line doctors being considered. The desire for training remained high, as 85.2% of second-line doctors have received either a university diploma and/or CPD training in NDDs.

**Table 2:** Type of NDD training based on the level of care.

Type of training					
	Not involved	Level 1	Level 2	Level 3	Total
University diploma on specific disorders	1	12.1% (18)	40% (84)	100% (4)	28.6% (107)
CPD training	30% (3)	57.7% (86)	74.3% (156)	75% (3)	66.3% (248)
University diploma and/or CPD	36.4% (4)	61.1% (91)	85.2% (179)	100% (4)	74.3% (278)
Training from a Training insurance fund	(0)	18.7% (28)	35.7% (75)	33.3% (1)	27.8% (104)
Total	11	149	210	4	374

Percentage based on the level of care of the respondent.

### Activity and methylphenidate prescription

The activity of private practice doctors taking part in the survey in 2023 covered between 48.7% and 53.6% of the expected annual diagnostic needs for complex NDDs at the national level (Table 3). As for follow-up consultations for NDDs, the private practice doctors taking part in the survey reported between 55,000 and 60,000 consultations per year, which covered between 24% and 26.4% of the estimated annual needs in France for childhood NDDs.

**Table 3:** Diagnoses and yearly follow-ups stated in the study based on the level of care in NDDs.

	Number of NDD diagnoses per year (based on 10 months of work)	Number of NDD diagnoses per year (based on 11 months of work)	Number of NDD follow-up consultations per year over 10 months	Number of NDD follow-up consultations per year over 11 months
Not trained	130	143	150	165
Level 1	5,525	6,077	6,700	7,370
Level 2	21,330	23,463	46,035	50,638
Level 3	1,100	1,210	2,400	2,640
Total	28,085	30,893	55,285	60,816
Expected total^a^	57,600	57,600	230,400	230,400
Percentage covered by private medical doctors^b^	48.7%	53.6%	24%	26.4%

^a^ Based on 812,000 yearly births in France (9) and a prevalence of 8%.

^b^ Based on a yearly incidence of 8% and an average of 4 follow-up consultations between 5 and 15 years old.

The initial prescription of Methylphenidate reported by the participants depended mainly on the level of care. Among untrained or level 1 doctors, 98.8% did not prescribe it, or only occasionally. Among second- and third-line doctors, most were still not very active in initiating treatment (75.1%), while a minority of doctors frequently prescribed it (24.9%) (Table 4).

**Table 4:** Initial prescription of methylphenidate declared by respondents in 2022.

Category of medical practitioner	No prescription	At most once per month	At most once per week	More than once per week	Total
Number of prescriptions declared in 2022 average (SD)	0	4.3 (2.9)	21.9 (9.1)	96.5 (44.4)	
Number of medical doctors	177	140	37	20	374
Number of responding medical doctors by level of care: non trained/1/2/3	10/110/57/0	1/37/102/0	0/2/35/0	0/0/16/4	11/149/210/5
Number of initial prescriptions declared in 2022	0	604	810	1930	3,344

## Discussion

This survey demonstrates that healthcare organization may involve private practice physicians in the care pathway for children with specific developmental and learning disorders, and more broadly for neurodevelopmental disorders, and is not restricted to the public sector.

### Demographics of private practice physicians in France for NDDs

**Figure 1:**
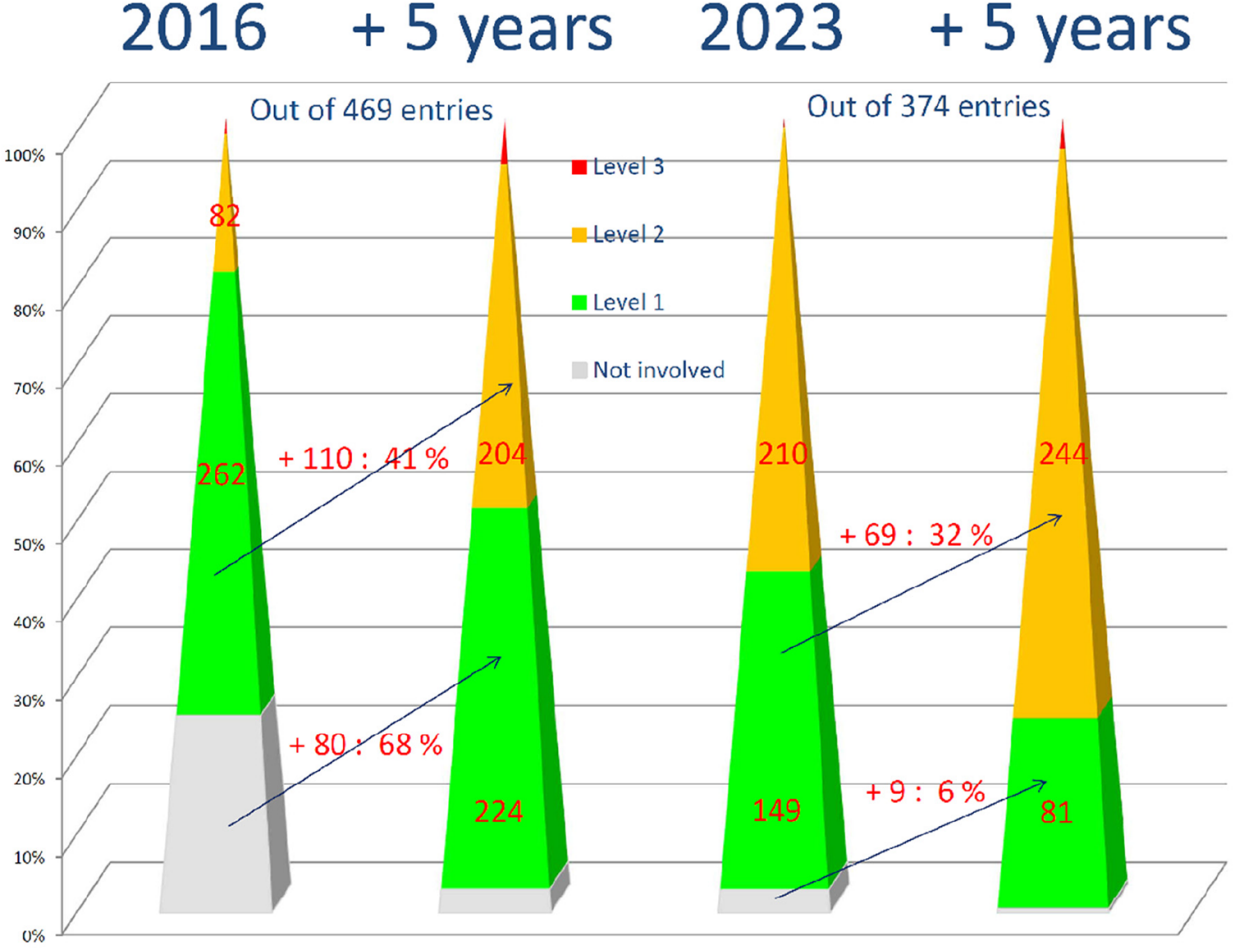
Distribution respondents in their respective level of care in 2016 (1) and in 2023 (data from this study) and estimated in 5 years. Caption: the number of physicians who took part in the survey based on their level of care in 2016 (1), in the current period (2023) and in 5 years. Between the pyramids: the number of respondents who changed their level of practice on NDDs numbers and percentage. In gray: non-involved physicians, in green first-level physicians, in yellow second-level physicians, and in red third-level physicians.

The data gathered in 2017 had predicted an increase in the skills of private practice physicians within 5 years thanks to the development of training (1). The situation observed by the 2022 survey confirms this projection. This makes the projection for 2,028 summarized in Figure 1 credible. Therefore, we can estimate that despite retirements, the population of second-line private practice physicians will count at least 250 physicians in France at that time. This 20% increase in the workforce of second-line private practice physicians, with constant activity, should allow for the diagnosis of between 58% and 63% of the annual needs for NDDs within 5 years. This situation regarding the commitment of physicians, and of pediatricians, is encouraging, not to mention the effects of the new internship reform and the official recognition of neuropediatrics, which will thus complement the demographics of second-line private practice physicians for NDDs.

Our data also provides encouraging figures as compared to the dramatic decrease in the demography of child psychiatrists in the public sector in France (21): future health policies will be able to consider this emerging care offer of private practice MDs and redefine the roles of respectively emerging developmental-behavioral medicine, and child and adolescent mental health structures.

Financial concerns may, however, influence this development. In France, two sectors of practice are open to the choice of the MDs, depending on their qualification. Fixed prices (as resulting from negotiations with the French “Sécurité Sociale”) open reimbursement by the social security and complementary health insurances but restrains considerably the ability to perform long medical evaluations: this is the regulated sector, also called in France, “Secteur 1”. For instance, the regular fee for a pediatric visit is 31 € (33 US $), while a long visit using neurocognitive tests applied by the MD does not exceed 69 € (73 US$). Conversely, “free sector” (secteur 2) allows the MD to determine their fees, allowing a time-consuming practice to remain economically viable, but engaging social and financial inequities in healthcare access, as these fees are usually not covered by the “Securité Sociale”, while usually being reimbursed by voluntary health insurances. Therefore, we included in our regional experiment “Parcours de Santé TSLA Occitanie” a fixed medical package for all patients included, allowing both families to benefit from a complete level and process of expertise, without limitation in the financial balance of the MDs. This was one of the packages experienced in region Occitanie and proposed for a national extension at the beginning of 2024.

Our survey stated that specialized medical activity in NDD patients is reduced due to insufficient fees compared to the length of consultations, except for doctors in sector 2, or those benefiting from specific care financing packages (as exists in the TSLA Occitanie healthcare pathway or in some networks for SpLD). It is essential to address this financial aspect in the future if we want to achieve 100% of the goals for this care pathway at the national level.

### Training of private practice physicians for NDD in France

The involvement of untrained physicians towards first-line practice remains an objective and requires a training plan, notably engaged by AFPA for the past 25 years.

This training for first-line practice can consist of two parts:

• On the one hand, basic training on the different disorders involved (oral language, praxis/visuospatial, attention, written language, mathematical cognition, social skills), allowing for identification, diagnosis, initial monitoring of simple situations and referral to appropriate structures.
• On the other hand, training in care coordination. This requires knowledge of the role of different paramedical professionals in assessments and re-education and the interpretation of speech therapy and psychomotor/occupational therapy assessments. These two training components are currently provided by Professional Continuing Development (PCD) training and through online training tools made available by the inter-ministerial delegation for autism within the neurodevelopmental disorders (22).

Training for second-line practice relies now on a rich offer, including in 2023, 15 University diplomas (23), most often supplemented in practical terms by continuous training provided by those involved within the national agency for CPD (ANDPC), or SpLD or NDD networks. The AFPA also provides training for private practice physicians, contributing to the increase in the skills of physicians towards a second-line level: 14 modules (with a total of 156 h), deliverable over a period of several years, accredited by the ANDPC or the training insurance fund (Fonds d'Assurance Formation, FAF).

Access to the first prescription of Methylphenidate has evolved significantly since September 2021, when the hospital initial prescription requirement was lifted, allowing pediatricians, psychiatrists, and neurologists in private practice to perform this first prescription. This evolution in French regulations allowed an improvement in access to pharmacological treatment with Methylphenidate in France in 2022. Prevalence data and access to prescription in Western countries (excluding the USA and Iceland) indicate a frequency of prescription between 1.8% and 2.2% among children aged 6–15 years old (19, 20), which, based on an average annual number of births of 812,000 between 2007 and 2017 (18), allows for an estimate of the needs for first prescriptions in this age group to be 16,240 per year in France. In 2022, the lifting of the hospital initial prescription requirement enabled private practice doctors to cover 20.5% of national needs. It can be estimated that in 5 years, with the improvement of the training of private practice doctors, about 50% of the needs will be covered by private practice MDs, enabling universal access to stimulant therapy for child and adolescent ADHD in France. Interestingly, our recently published research established that the previously reported opposition of MDs to the prescription of stimulant therapy is no longer an obstacle in two large regions of France (14).

### What we have learned from other healthcare models or organization

The benefits of coordinated care pathways in NDD have been the subject of research studies around the world, mainly focused on autism and ADHD. In the Netherlands, the Tornado program is attempting to evaluate the benefit of access to a coordinated care pathway that includes primary care practitioners for ADHD patients, but no results have been published yet (24). In Scotland, the Dundee ADHD Clinical Care Pathway proposes a model of organization of care for ADHD that includes delegation of tasks to nurses in follow-up care, including the prescription renewal (25). In the United States, the involvement of primary care pediatricians in learning disorders and ADHD is at a level comparable to the data obtained in France from two surveys conducted ten years apart among members of the American Academy of Pediatrics between 2004 and 2013. Engagement for learning disorders increased from 51.4% to 63.5% for first-line “inquire or screen” and from 7.9% to 18.3% for second-line “treat/manage/co-manage”, but reached 57.6% for ADHD (26). Systematic reviews of integrated care models in ADHD have established their potential to improve access and accessibility of care but with concerns on the financial provisions (27), explaining some of the social gradients of health services and health-related quality of life (28).

Regarding autism, historical healthcare pathways (29) evolve towards neurodevelopmental pathways, as it has been described by researchers from Scotland, Sweden and the UK (30, 32), improving the diagnostic procedures and the comorbidities involved.

From the USA, a federally funded initiative on coordinated evaluation and care has succeeded in covering the majority of states, together with evaluations of the efficiency of the training programs through the Leadership Education in Neurodevelopmental and related Disabilities (LEND) programs across the United States (33).

Training of MDs may also be enriched by a specific certification in developmental-behavioral medicine, as it is already structured for pediatricians in the USA and Canada (34), which could be extended in France to other specialties like general medicine or child and adolescent mental health, as part of the second-tier level in France is already covered by these professionals. Similarly, the federation of a dedicated professional society devoted to this field as it is structured through the Developmental-Behavioral Pediatrics Research Network (DBPNet) could help to enhance access to care for families, exchanges between researchers and the structure of both regional and national networks.

### How could the French model enrich other countries

Our present data confirm the involvement of Second tiers MDs involved in NDD, as a part of the nationwide two-decade effort to build a structured healthcare policy ensuring universal access to a level of care adapted to the complexity of each individual patient.

It relies on several steps, including a continuous effort of training for MDs, with programs adapted to the level of care of in which the professional wishes to be involved, the design by the national social security or the private insurances of a full level of coverage of fees adapted to the time required for specialized evaluations, regardless of the administrative type of organization. Flexibility in national and regional health authorities is also essential. Indeed, as experts in our National Health authority, we chose to focus on the respect of neurocognitive concepts, to systematically favor a multidisciplinary approach together with a systematic organization of multidisciplinary team meeting, as they were established initially in oncology, and are part of the recommendations on SpLD (9, 35).

Another process of evolution is the systematic training of tier 1 and tier 2 professionals to the various NDDs, and to break down barriers between organizations initially devoted to a single disorder, such as autism, SpLD or ADHD. As for the patients, these borders represent an obstacle to an accurate definition of their needs. Expert teams (tier 3) might be still mandatory but reserved for highly complex situations.

In France, a major progress has been recently decided by the government, through a national strategy for NDD, by providing a concerted and universal approach on various aspects of the needs in education, care and family (36). In this perspective, we have been demonstrating that opening up the healthcare system to private practitioners might also take into account both the increase in demand as encountered in most countries, and the reduction in offer, as for instance the paucity of child psychiatrists in France and in many other countries (21).

At the regional level, our research has also established that a coordinated effort involving Regional Health authorities, Health insurances, unions of MDs, and a network of the different professionals may allow for the development of a coordinated healthcare pathway.

The TSLA Occitanie health pathway, an experimental Article 51 program led by Occitadys, which follows the recommendations of the French HAS, provides elements for its generalization beyond 2024 (18). The deployment of 15 secondary care centers in Occitanie is already complete, covering all 13 departments and providing a response for first- and second-line diagnoses fully financed by the health insurance, with no out-of-pocket costs for families (for more than 3,500 children per year). One of the innovative aspects of this project is to make long consultations with second-line physicians affordable for families. Indeed, they are paid through a fixed-price package of €300, without any surcharge or financial participation from families, covering all diagnostic and restitution consultations and medical procedures. This package, like all other TSLA Occitanie packages, is awarded regardless of the physician's mode of practice: private practice or employed in a hospital or medico-social institution. However, it remains up to the technical committee for innovation in health (CTIS), the strategic council for innovation in health (CSIS) and the political power to decide on whether to make it permanent and available in the whole country after the end of the experiment, thus reducing regional disparities. The development of the new national strategy for neurodevelopmental disorders must include the healthcare offer of second-line private practice physicians, who already cover half of the national needs, thus representing an important component of the medical offer for the patients and their families.

The main study limitations rely first on the descriptive and voluntary nature of our work, potentially leading to an underestimation of the total number of private practitioners currently involved in NDD in France. This challenge persists due to the absence of an official subspecialty on NDDs in France, unlike in Canada and the USA, where there exist established networks and medical qualifications for developmental and behavioural pediatrics (34).

The second study limitation is linked to the inaccuracies in the classification of medical practices (employed, private, or mixed) from the National Health Insurance Database (SNDS), rendering the data unusable for this purpose of analysing the initiation of stimulant therapy in France according to the sector of the MDs (to be published). Similarly, despite our efforts, we encountered difficulties accessing national data on the annual activities of public healthcare services, including those in the social and healthcare sectors responsible for diagnosing and providing follow-up care for children with NDD in France. Consequently, our dataset remains restricted to the private sector.

## Conclusions

A comprehensive strategy aimed at enhancing both the proficiency and quantity of second-line medical doctors (MDs) necessitates thoughtful consideration of various aspects. These include factors such as the initial training provided during residency, postgraduate training, continuous medical education for practitioners, the adjustment of medical fees, and the structuring of second and third lines of expertise or specialized networks. Such initiatives serve as a crucial support system for first-line professionals.

In the pursuit of establishing a tiered organizational framework for developmental disorders that is easily accessible and transcends barriers between medical specialties, our data underscores the need to move beyond the conflicts inherent in separate healthcare pathways. Instead, we advocate for a shift towards an integrated Neurodevelopmental Disorders (NDD) pathway.

Second-line MDs specializing in NDD occupy a pivotal role, serving as a bridge between first-line MDs and families. Our experience in the Occitanie region, in collaboration with other stakeholders from pediatric services, mental health services, and medico-social providers, highlights the effectiveness of this approach. Consequently, the nationwide implementation of this model stands to benefit from our data-driven insights, facilitated by an increasing number of professionals contributing to a harmonious system that integrates both private practitioners and employed MDs.

## Data Availability

The raw data supporting the conclusions of this article will be made available by the authors, without undue reservation.
